# The effects of cement fixation on survival in elderly patients with hip hemiarthroplasty: a nationwide cohort study

**DOI:** 10.1186/s12891-019-3013-2

**Published:** 2019-12-27

**Authors:** Ming-Che Tsai, Yee-Yung Ng, Wei-Ming Chen, Shang-Wen Tsai, Shiao-Chi Wu

**Affiliations:** 1 Institute of Health and Welfare Policy, School of Medicine, National Yang-Ming University, Taipei, Taiwan; 2 Department of Medicine, Fu Jen Catholic University Hospital, Fu Jen Catholic University, New Taipei City, Taiwan; 3 Department of Orthopaedics, Taipei Veterans General Hospital, School of Medicine, National Yang-Ming University, Taipei, Taiwan; 4 Department of Orthopaedics, Taipei Veterans General Hospital, Taipei, Taiwan; 5 Institute of Health and Welfare Policy, College of Medicine, National Yang-Ming University, No.155, Sec.2, Linong Street, Taipei, 11221 Taiwan, Republic of China

**Keywords:** Cement, Elderly patient, Hip hemiarthroplasty, Postoperative survival

## Abstract

**Background:**

Hip hemiarthroplasty (HHA) is a common treatment for hip fractures in the elderly population. Because of the fatal effects of bone cement implantation syndrome, the safety of cement utilization to enhance implant firmness in the femur is controversial. The aim of this study was to investigate the postoperative survival of elderly patients receiving HHA with and without cement fixation.

**Methods:**

Claim data from the National Health Insurance Database and the National Register of Deaths Database were used for analysis in this retrospective cohort study. From 2008 to 2014, 25,862 patients aged 80 years or older treated with hip hemiarthroplasty were included in the analysis. A Cox proportional risk model was used to analyse the effects of cement utilization on postoperative mortality.

**Results:**

The cemented group had a significantly higher mortality risk than the non-cemented group within 7, 30, 180 days and 1 year after the operation. The effect of bone cement on postoperative mortality was significantly stronger within 7 days than within 30, 180 days and 1 year. In addition, the male gender, age > 85 years and higher score on the Charlson Comorbidity Index were also risk factors for mortality (*p* < 0.05). Patients who received HHA in lower-volume hospitals had higher mortality rates within 180 days and 1 year than those in higher-volume hospitals. Compared with patients who were operated on by high-volume surgeons, those who received surgery performed by lower-volume surgeons were more likely to die within 30 days (aHR = 1.22), 180 days (aHR = 1.16) and 1 year (aHR = 1.19), respectively.

**Conclusions:**

The postoperative mortality rate of elderly patients undergoing HHA was significantly higher in the cemented group than in the non-cemented group.

## Background

In light of the increased number of hip fractures in the elderly above 80 years old, hip arthroplasty is an important issue in this population. Compared with total hip arthroplasty (THA), hip hemiarthroplasty (HHA) is a surgical procedure that is simpler and cheaper and yields fewer postoperative complications [1]; moreover, HHA is suitable for patients with displaced femoral neck fractures [2, 3].

Although uncemented HHA has advantages such as a shorter operation time and less bleeding during the procedure [4], cemented HHA might still be performed based on the patient’s age and bone mineral density, the surgeon’s training, and the management of rehabilitation protocols [5, 6]. Nonetheless, the toxic effects of bone cement might increase the risk of cardiopulmonary collapse [4, 7], and fatal bone cement implantation syndrome (BCIS) [8]. However, there is no consensus in recent studies on the postoperative mortality of patients who received bone cement [9–15]. The lack of consensus may be related to the fact that most of the previous studies were limited to a single institution, had a small sample size or used limited variables considered in database research. Therefore, the purpose of this study was to use a nationwide claims database to investigate the postoperative survival of elderly patients undergoing HHA with different fixation methods.

## Methods

### Settings

This retrospective cohort study used claims data during the period of 1 January 2007–31 December 2015 from the National Health Insurance Database (NHID) and the National Register of Deaths Database (NRDD) for analysis. Based on the NHID, patients who were aged 80 years or older and diagnosed with femoral neck fracture (International Classification of Diseases, Ninth Revision, Clinical Modification (ICD-9-CM) codes: 820.xx) between January 1, 2008, and December 31, 2014, and were treated with HHA (ICD-9-CM procedure codes: 81.52) were eligible for inclusion (*n* = 26,247). The patients who underwent THA (ICD-9-CM procedure codes: 81.51), HHA, or revision of hip replacement (ICD-9-CM procedure codes: 81.53) during hospitalization (*n* = 24) or one year prior to hospitalization (*n* = 361) were excluded from this study. Finally, 25,862 cases were included in the analysis.

### Data collection

The National Health Insurance programme in Taiwan has included over 99.7% of the Taiwanese population since 2004 and provides universal, compulsory coverage with low co-payments to minimize the economic barrier to care for patients needing inpatient, outpatient, prescription, and other services. The NHID contains each patient’s demographic characteristics, medical treatment location and detailed records of outpatient visits, hospital admissions, and emergency department (ED) visits, including diagnoses, procedures, medications, providers, and expenses [16]. The NRDD monitors the completeness and accuracy of death registration data retrieved from the Ministry of Health and Welfare, and the data contained age, sex, date of death, and causes of death. Both databases are encrypted, patient data are de-identified, and the database is monitored for completeness and accuracy by Taiwan’s Ministry of Health and Welfare. The authors analysed data at the Health and Welfare Data Science Centre, which is an independent workplace managed by Taiwan’s Ministry of Health and Welfare.

The dependent variable of this study was all-cause mortality within 1 year after the operation. The duration from the first day of hospitalization to the day of death was defined as the overall survival time. Subjects who were still alive after 1 year of follow-up were censored.

The fixation method (cemented or uncemented) was the main independent variable in this study and was identified by payment codes from the Taiwan NHI Medical Service Benefits and Payment Criteria [17]. Other covariates included characteristics of patients and healthcare providers. The patients’ characteristics included sex, age group (80–84 years, 85–89 years, ≥90 years), insurance status (≥840 USD, < 840 USD) [18], Charlson comorbidity index (CCI) status (CCI = 0, CCI ≥ 1) during the last year before the operation [19–21] and type of head used in the operation (unipolar hemiarthroplasty (UHA) or bipolar hemiarthroplasty (BHA)) [22–25]. The insurance premiums were determined by the enrolee’s income and therefore were a proxy for income status [26]. Insurance status was classified into two groups according to the median of the insurance premiums.

The providers’ characteristics included the type of hospital (private (including corporate) or public hospital) [27, 28], hospital accreditation level (medical centre, non-medical centre) [28], the annual volumes of hip replacement for the hospital [29–33] and surgeon [29, 30, 34]. Annual volumes of hip replacement were classified into two groups according to the median operation case number among all the providers/surgeons.

### Statistical analysis

Pearson’s χ2 test was used to compare characteristics between the study participants who received cement and those who did not. The log-rank test was used to compare the mortality rates of different time intervals (7, 30, 180 days and 1 year from the operation) between participants receiving cement and non-cement HHA. To assess the one-year mortality risk factors of different fixation methods, we fitted a Cox proportional hazard regression with covariates including sex, age group, insurance status, CCI score, hospital type, hospital level, hospital volume, and surgeon volume. We also investigated multicollinearity by the variance inflation factor (VIF) using regression analysis. Because the VIF of each coefficient was less than 5, we presumed that the effect of correlation among the independent variables was not enough to distort the estimation. For data management and statistical analysis, we used the statistical software SAS 9.4 (SAS Institute, Inc., Cary, North Carolina, USA).

### Ethics and conflict of interest

This study was approved by the Institutional Review Board of National Yang-Ming University in Taiwan (approval number YM105043E-3). The authors declare no any financial and non-financial competing interests.

## Results

### The basic characteristics of patients who received HHA

In total, 47.8% (12,364) of patients received cemented HHA. The percentages of female patients, patients with age ≥ 90 years, patients with lower insurance premiums, patients receiving UHA, patients treated at higher-volume hospitals, and patients operated on by higher-volume surgeons were significantly higher in the cemented HHA group than in the uncemented HHA group. The CCI score was not significantly different between the two groups (Table 1).

**Table 1:** Description of elderly patients undergoing hip hemiarthroplasty

	Total		Uncemented		Cemented		P-value
	(n = 25,862)		(n = 13,498)		(n = 12,364)		
	N	%	N	%	N	%	
Enrolee characteristics							
Sex							< 0.001
Male	9,123	35.3	4,978	54.6	4,145	45.4	
Female	16,739	64.7	8,520	50.9	8,219	49.1	
Age group							< 0.001
80–84 years	12,284	47.5	6,728	54.8	5,556	45.2	
85–89 years	8,950	34.6	4,557	50.9	4,393	49.1	
≥ 90 years	4,628	17.9	2,213	47.8	2,415	52.2	
Insurance status							< 0.001
≥ US $840	14,509	56.1	7,903	54.5	6,606	45.5	
< US $840	11,353	43.9	5,595	49.3	5,758	50.7	
Comorbidity							
CCI score							0.143
CCI = 0	5,557	21.5	2,852	51.3	2,705	48.7	
CCI ≥ 1	20,305	78.5	10,646	52.4	9,659	47.6	
Type of head							< 0.001
Unipolar HA	11,265	43.6	3,627	32.2	7,638	67.8	
Bipolar HA	14,597	56.4	9,871	67.6	4,726	32.4	
Provider characteristics							
Hospital type							< 0.001
Public	9,226	35.7	4,531	49.1	4,695	50.9	
Private	16,636	64.3	8,967	53.9	7,669	46.1	
Hospital level							< 0.001
Medical centre	7,679	29.7	3,370	43.9	4,309	56.1	
Non-medical centre	18,183	70.3	10,128	55.7	8,055	44.3	
Hospital volume							< 0.001
High (volume > 120)	10,367	40.1	4,646	44.8	5,721	55.2	
Low (volume ≤ 120)	15,495	59.9	8,852	57.1	6,643	42.9	
Surgeon volume							< 0.001
High (volume > 21)	9,258	35.8	4,173	45.1	5,085	54.9	
Low (volume ≤ 21)	16,604	64.2	9,325	56.2	7,279	43.8	

Note: Distribution among groups was analysed by the *x*^*2*^-square test

### Postoperative mortality of elderly patients undergoing HHA

As shown in Table 2, the overall postoperative mortality rates within 7, 30, 180 days and 1 year among senior patients who received HHA were 0.6, 3.1, 11.2, and 17.3%, respectively. The mortality rates at 7 days (0.7% vs 0.4%, *p* < 0.01), 30 days (3.5% vs 2.7%, *p* < 0.01), 180 days (11.8% vs 10.7%, *p* < 0.01) and 1 year (18.0% vs 16.7%, *p* < 0.01) were significantly higher in the cement group than in the non-cement group.

**Table 2:** Postoperative mortality of elderly patients undergoing hip hemiarthroplasty

	Total	7-days		30-days		180-days		1-year	
	N	%	*P*	%	*P*	%	*P*	%	*P*
All	25,862	0.6		3.1		11.2		17.3	
Fixation method			0.001		< 0.001		0.003		0.005
Cemented	12,364	0.7		3.5		11.8		18.0	
Uncemented	13,498	0.4		2.7		10.7		16.7	
Gender			0.013		< 0.001		< 0.001		< 0.001
Male	9,123	0.7		4.2		15.2		23.3	
Female	16,739	0.5		2.5		9.1		14.1	
Age Group			0.002		< 0.001		< 0.001		< 0.001
80–84 years	12,284	0.4		2.2		8.8		14.2	
85–89 years	8,950	0.7		3.3		11.9		18.1	
≥ 90 years	4,628	0.8		4.9		16.2		24.1	
Insurance status			0.468		0.054		0.220		0.066
≥ US $840	14,509	0.6		3.3		11.4		17.7	
< US $840	11,353	0.5		2.9		10.9		16.8	
CCI score			0.229		< 0.001		< 0.001		< 0.001
CCI = 0	5,557	0.5		2.2		12.2		18.7	
CCI ≥ 1	20,305	0.6		3.3		15.2		23.3	
Type of head			0.131		0.417		0.365		0.033
UHA	11,265	0.7		3.2		11.4		17.9	
BHA	14,597	0.5		3.0		11.1		16.9	
Hospital type			0.842		0.584		0.328		0.022
Public	9,226	0.6		3.2		11.5		18.0	
Private	16,636	0.6		3.1		11.1		16.9	
Hospital level			0.964		0.213		0.003		0.004
Medical centre	7,679	0.6		2.9		10.3		16.3	
Non-medical centre	18,183	0.6		3.2		11.6		17.7	
Hospital volume			0.428		0.184		< 0.001		< 0.001
High (> 120)	10,367	0.5		2.9		10.2		16.0	
Low (≤120)	15,495	0.6		3.2		11.9		18.2	
Surgeon volume			0.360		0.015		< 0.001		< 0.001
High (> 21)	9,258	0.5		2.7		10.2		15.6	
Low (≤21)	16,604	0.6		3.3		11.8		18.3	

Note: Distribution among groups was analysed by the log-rank test

### Risk factors for mortality in elderly patients receiving HHA

Tables 3 and 4 present the crude and adjusted hazard ratios of risk factors of mortality within 7, 30, 180 days and 1 year. The patients who received bone cement had a significantly higher mortality risk than those who did not receive bone cement within 7, 30, 180 days and 1 year (aHR = 1.8, 95% CI = 1.23–2.51, *p* < 0.01; aHR = 1.4, 95% CI = 1.17–1.58, *p* < 0.01; aHR = 1.2, 95% CI = 1.08–1.26, *p* < 0.01; aHR = 1.1, 95% CI = 1.04–1.18, *p* < 0.01), respectively. The type of head and insurance status were not risk factors for mortality (*p* > 0.05). Male gender and age > 85 years were risk factors for mortality within 7, 30, 180 days and 1 year (*p* < 0.05). CCI scores were identified as a significant risk factor for 30 days, 180 days and 1-year mortality (*p* < 0.05). After adjustment, hospital type and hospital level were not risk factors for mortality (*p* > 0.05) (Table 4). Patients who received HHA at lower-volume hospitals were 1.16 and 1.15 times more likely to die within 180 days and 1 year than those who received HHA at high-volume hospitals (95% CI = 1.05–1.29, *p* < 0.01; 95% CI = 1.06–1.24, *p* < 0.01), respectively (Table 4). Compared with patients who were operated on by high-volume surgeons, those who were operated on by lower-volume surgeons were more likely to die within 30 days (aHR = 1.22, 95% CI = 1.05–1.42, *p* < 0.05), 180 days (aHR = 1.16, 95% CI = 1.07–1.25, *p* < 0.01) and 1 year (aHR = 1.19, 95% CI = 1.12–1.27, *p* < 0.01). The effect of bone cement on postoperative mortality was significantly stronger within 7 days than within 30, 180 days and 1 year (Table 5).

**Table 3:** Risk factors for 7-day, 30-day, 180-day and 1-year mortality using Cox regression analysis with crude hazard ratios (cHRs) and 95% confidence intervals (CIs)

		7 days			30 days			180 days			1 year	
	cHR	(95% CI)	*P*	cHR	(95% CI)	*P*	cHR	(95% CI)	*P*	cHR	(95% CI)	*P*
**Fixation method (Ref: Uncemented)**												
Cemented	1.72	1.23–2.39	0.001	1.29	1.12–1.48	< 0.001	1.12	1.04–1.20	0.003	1.08	1.02–1.15	0.007
Gender (Ref: Female)												
Male	1.50	1.09–2.07	0.014	1.67	1.45–1.92	< 0.001	1.73	1.61–1.86	< 0.001	1.76	1.66–1.86	< 0.001
Age group (Ref: 80–84 years)												
85–89 years	1.68	1.15–2.44	0.007	1.48	1.26–1.74	< 0.001	1.39	1.27–1.51	< 0.001	1.30	1.22–1.39	< 0.001
≥ 90 years	2.02	1.33–3.08	0.001	2.23	1.87–2.66	< 0.001	1.94	1.76–2.13	< 0.001	1.82	1.69–1.96	< 0.001
Insurance status (Ref: ≥US $840)												
< US $840	0.89	0.64–1.23	0.468	0.87	0.76–1.00	0.054	0.96	0.89–1.03	0.221	0.95	0.90–1.01	0.077
CCI score (Ref: CCI = 0)												
CCI ≥ 1	1.30	0.85–1.98	0.231	1.53	1.26–1.86	< 0.001	1.62	1.46–1.79	< 0.001	1.57	1.45–1.70	< 0.001
Type of head (Ref: BHA)												
UHA	1.28	0.93–1.11	0.133	1.06	0.92–1.22	0.416	1.04	0.96–1.11	0.364	1.07	1.01–1.13	0.034
Hospital type (Ref: Private)												
Public	0.97	0.69–1.35	0.843	1.04	0.90–1.20	0.582	1.04	0.96–1.12	0.326	1.07	1.01–1.13	0.034
Hospital level (Ref: Medical centre)												
Non-medical centre	1.01	0.71–1.43	0.964	1.10	0.95–1.29	0.214	1.13	1.05–1.23	0.003	1.11	1.04–1.18	0.002
Hospital volume (Ref: High)												
Low	1.14	0.82–1.60	0.428	1.10	0.96–1.27	0.185	1.18	1.10–1.28	< 0.001	1.15	1.09–1.23	< 0.001
Surgeon volume (Ref: High)												
Low	1.17	0.83–1.60	0.362	1.20	1.04–1.40	0.015	1.17	1.08–1.26	< 0.001	1.20	1.13–1.27	< 0.001

**Table 4:** Risk factors for 7-day, 30-day, 180-day and 1-year mortality using Cox regression analysis with adjusted hazard ratios (aHRs) and 95% confidence intervals (CIs)

		7-days			30-days			180-days			1-year	
	aHR	(95% CI)	*P*	aHR	(95% CI)	*P*	aHR	(95% CI)	*P*	aHR	(95% CI)	*P*
Fixation method (Ref: Uncemented)												
Cemented	1.76	1.23–2.51	0.002	1.36	1.17–1.58	< 0.001	1.16	1.08–1.26	< 0.001	1.11	1.04–1.18	0.001
Gender (Ref: Female)												
Male	1.52	1.10–2.12	0.012	1.68	1.46–1.93	< 0.001	1.75	1.62–1.88	< 0.001	1.75	1.65–1.86	< 0.001
Age group (Ref: 80–84 years)												
85–89 years	1.64	1.13–2.39	0.009	1.47	1.25–1.74	< 0.001	1.39	1.28–1.51	< 0.001	1.31	1.23–1.40	< 0.001
≥ 90 years	2.02	1.32–3.08	0.001	2.31	1.94–2.75	< 0.001	2.03	1.85–2.23	< 0.001	1.89	1.76–2.04	< 0.001
Insurance status (Ref: ≥US $840)												
< US $840	0.92	0.65–1.29	0.612	0.93	0.81–1.08	0.345	1.04	0.97–1.13	0.271	1.06	0.99–1.12	0.077
CCI score (Ref: CCI = 0)												
CCI ≥ 1	1.31	0.86–2.01	0.211	1.55	1.28–1.88	< 0.001	1.62	1.46–1.80	< 0.001	1.55	1.44–1.68	< 0.001
Type of head (Ref: BHA)												
UHA	1.03	0.73–1.46	0.855	0.93	0.81–1.09	0.373	0.97	0.90–1.05	0.435	0.99	0.93–1.05	0.703
Hospital type (Ref: Private)												
Public	0.88	0.62–1.24	0.461	0.94	0.81–1.10	0.446	0.97	0.90–1.05	0.437	1.00	0.94–1.06	0.895
Hospital level (Ref: Medical centre)												
Non-medical centre	0.89	0.55–1.46	0.652	1.10	0.89–1.35	0.388	1.03	0.92–1.15	0.578	1.00	0.92–1.09	0.973
Hospital volume (Ref: High)												
Low	1.28	0.80–2.05	0.296	1.05	0.87–1.28	0.596	1.16	1.05–1.29	0.004	1.15	1.06–1.24	0.001
Surgeon volume (Ref: High)												
Low	1.22	0.85–1.73	0.280	1.22	1.05–1.42	0.011	1.16	1.07–1.25	< 0.001	1.19	1.12–1.27	< 0.001

**Table 5:** Effect of bone cement on postoperative mortality in elderly survivors undergoing hip arthroplasty

	7 days to 1 year			30 days to 1 year			180 days to 1 year		
	Event	*%*	*P*	Event	*%*	*P*	Event	*%*	*P*
All	4,327	16.8		3,675	14.7		1,577	6.9	
Fixation method			0.024			0.157			0.452
Cemented	2,130	17.4		1,788	15.0		763	7.0	
Uncemented	21,97	16.3		1,887	14.4		814	6.8	

Note: 1. the survivors in the first 7, 30, 180 days were followed up to 1 year for postoperative mortality

2. Distribution among groups was analysed by the log-rank test

## Discussion

To the best of our knowledge, this is the first study using nationwide claim data to evaluate the effect of the HHA fixation method on survival in elderly patients. It is worth noting that the cemented group had a significantly higher mortality risk than the non-cemented group within 7, 30, 180 days and 1 year after the operation. The postoperative mortality effect of bone cement was significantly decreased after 7 days (Tables 3, 4 and 5). This finding might explain previous studies revealing that cement has different impacts according to different postoperative follow-up periods [9, 12]. The cement group had significantly higher mortality within 7 days but no difference after 30 days (Table 5). In other words, the mortality risk correlated with the usage of cement mainly occurred in the first 7 days after surgery. Thus, the higher mortality in the cement group might be associated with potentially fatal complications of BCIS [35, 36].

The previous literature showed that the provider’s experience and volume remained an important factor for postoperative survival in different kinds of operations, including eye surgery, coronary artery bypass graft, HHA and total hip replacement [29–34, 37, 38]. These studies further support our findings that patients treated at high-volume hospitals and by high-volume surgeons had a lower risk. Hospitals and surgeons with a high volume of cases may have more experience to avoid mistakes, resulting in fewer adverse consequences and complications [29–34].

In our study, male gender, older age and higher score on the comorbidity index (CCI≧1) were the independent determinants of increased mortality, which were also consistent with other studies [11, 20, 22–24, 30, 39, 40]. Because the degree of biological ageing significantly affects changes in body tissue structure and dysfunction, older patients are prone to have medical complications and higher mortality rates. Patients with high CCI values may have various kinds of comorbidities or even more severe conditions, such as COPD [18, 40, 41], cognitive impairment [11, 18] and heart disease [18, 35, 40], which may affect postoperative mortality. The higher mortality rates in men than in women may be related to their lower ability of self-care, which may also result in the occurrence of more complications or even lead to death [41].

Although the large amount of administration data in the NHID could avoid sampling bias, the main purpose of the NHID is to apply for medical insurance expenditure. Clinical data such as clinical notes, operational procedures, disease severity, and radiological images were lacking as other administration datasets. Other potential risk factors of mortality, such as surgical time, surgical methods, material characteristics such as type of stem [42–44], BMI [45], walking ability [46], disease severity (11, 40), self-care ability and strength of family support [47, 48], may not be documented in the NHID. Many observational variables were adjusted in this study, and Taiwanese research [23] has revealed that the activities of daily living (ADL) and the American Society of Anesthesiologists (ASA) Classification scores have no significant effects on survival outcomes. This study can only be considered regarding the influence of medical care but cannot be extended to explain the factors related to family care and that may affect postoperative survivorship. However, our study used the characteristics of medical care providers, including hospitals and surgeons, as alternative variables to decrease the possible influence of the potential risk factors of mortality [11, 31, 46].

## Conclusion

The postoperative mortality in elderly patients with HHA was significantly higher in patients receiving cement, especially within 7 days. The higher mortality rates were also associated with higher CCI scores of the patient and lower operation volumes of the hospitals and surgeons.

## Data Availability

The data that support the findings of this study are available from the Ministry of Health and Welfare in Taiwan but restrictions apply to the availability of these data, which were used under license for the current study, and so are not publicly available. These databases were encrypted, de-identified and monitored for completeness and accuracy by the Ministry of Health and Welfare in Taiwan. Data are however available from the authors upon reasonable request and with permission of the Ministry of Health and Welfare.

## Abbreviations

95% CI: 95% Confidence interval
AHR: Adjusted hazard ratio
BCIS: Bone cement implantation syndrome
BHA: Bipolar hemiarthroplasty
CCI: Charlson comorbidity index
HHA: Hip hemiarthroplasty
ICD-9-CM: International Classification of Diseases, 9th revision, Clinical Modification
THA: Total hip arthroplasty
UHA: Unipolar hemiarthroplasty
